# Crystal structure of *rac*-(3aR*,9aS*)-4,4,4-tri­chloro-1,2,3,3a,4,9a-hexa­hydro-4λ^5^,9λ^4^-cyclo­penta­[4,5][1,3]tellurazolo[3,2-*a*]pyridine

**DOI:** 10.1107/S2056989015012311

**Published:** 2015-07-22

**Authors:** Rizvan K. Askerov, Julia M. Lukiyanova, Zhanna V. Matsulevich, Alexander V. Borisov, Victor N. Khrustalev

**Affiliations:** a Department of Chemistry, Baku State University, 23 Z. Khalilov St, Baku, AZ-1148, Azerbaijan; b R. E. Alekseev Nizhny Novgorod State Technical University, 24 Minin St, Nizhny Novgorod 603950, Russian Federation; c Inorganic Chemistry Department, Peoples’ Friendship University of Russia, 6 Miklukho-Maklay St, Moscow 117198, Russian Federation; d A. N. Nesmeyanov Institute of Organoelement Compounds of Russian Academy of Sciences, 28 Vavilov St, Moscow 119991, Russian Federation

**Keywords:** crystal structure, arenetellurium trihalide, Te⋯Cl inter­actions, C—H⋯Cl hydrogen bonding

## Abstract

The title compound, C_10_H_12_Cl_3_NTe, crystallizes with two crystallographically independent mol­ecules (*A* and *B*) in the asymmetric unit. In each case, the coordination around the Te atom is distorted square-pyramidal, with the equatorial plane composed of the three Cl atoms and the C atom of the pyridinium ring. The Te atom is displaced from the mean-square plane by 0.1926 (7) and 0.1981 (8) Å, in mol­ecules *A* and *B*, respectivly, away from the apical C atom. The bond lengths from the Te atom to the two Cl atoms arranged *trans* to each other [2.5009 (7)/2.5145 (7) and 2.5184 (7)/2.5220 (8) Å in mol­ecules *A* and *B*, respectivly] are substan­ti­ally shorter than the third Te—Cl distance [2.8786 (7) and 2.8763 (7) Å in mol­ecules *A* and *B*, respectivly]. The 1,3-tellurazole ring is almost planar (r.m.s. deviations of 0.042 and 0.045 Å in mol­ecules *A* and *B*, respectivly). The cyclopentane rings in both molecules *A* and *B* adopt envelope conformations with the carbon atom opposed to the (Te)C—C(N) bond as the flap. In the crystal, mol­ecules form centrosymmetric 2 + 2 associates *via* Te⋯Cl inter­actions [3.3993 (7) and 3.2030 (7) Å]. As a result of these secondary inter­actions, the Te atom attains a strongly distorted 5 + 1 octa­hedral environment. Further, the 2 + 2 associates are bound by weak C—H⋯Cl hydrogen bonds into a three–dimensional framework.

## Related literature   

For general background and synthesis, see: Petragnani & Stefani (2007 ▸); Borisov *et al.* (2013 ▸). For related compounds, see: Singh *et al.* (1990 ▸); Sundberg *et al.* (1994 ▸); Zukerman-Schpector *et al.* (2000 ▸); Kandasamy *et al.* (2003 ▸); Raghavendra *et al.* (2006 ▸); Dutton *et al.* (2009 ▸); Lee *et al.* (2010 ▸); Rakesh *et al.* (2012 ▸).
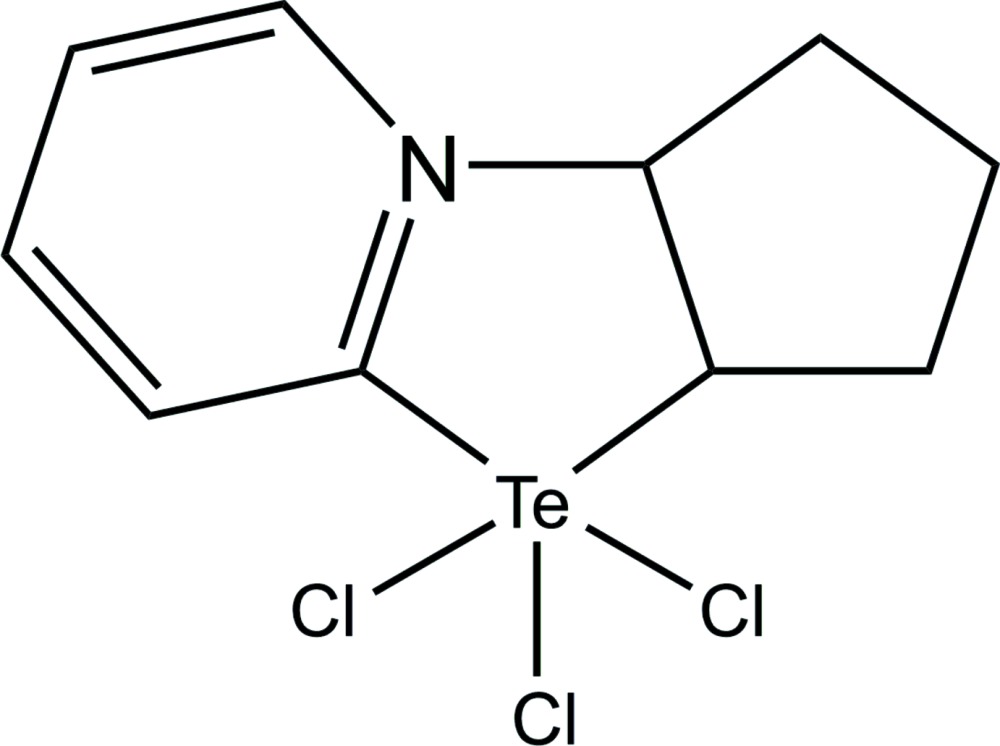


## Experimental   

### Crystal data  

C_10_H_12_Cl_3_NTe
*M* *_r_* = 380.16
Monoclinic, 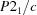

*a* = 14.3279 (6) Å
*b* = 11.2539 (5) Å
*c* = 16.2967 (7) Å
β = 94.546 (1)°
*V* = 2619.5 (2) Å^3^
*Z* = 8
Mo *K*α radiation
μ = 2.85 mm^−1^
*T* = 120 K
0.20 × 0.15 × 0.15 mm

### Data collection  

Bruker APEXII CCD diffractometer
Absorption correction: multi-scan (*SADABS*; Bruker, 2003 ▸) *T*_min_ = 0.595, *T*_max_ = 0.666
32448 measured reflections
7642 independent reflections
6535 reflections with *I* > 2σ(*I*)
*R*_int_ = 0.037

### Refinement  

*R*[*F* ^2^ > 2σ(*F* ^2^)] = 0.030
*wR*(*F* ^2^) = 0.059
*S* = 1.08
7642 reflections
271 parameters
H-atom parameters constrained
Δρ_max_ = 0.76 e Å^−3^
Δρ_min_ = −0.56 e Å^−3^

### 

Data collection: *APEX2* (Bruker, 2005 ▸); cell refinement: *SAINT* (Bruker, 2001 ▸); data reduction: *SAINT*; program(s) used to solve structure: *SHELXTL* (Sheldrick, 2008 ▸); program(s) used to refine structure: *SHELXTL*; molecular graphics: *SHELXTL*; software used to prepare material for publication: *SHELXTL*.

## Supplementary Material

Crystal structure: contains datablock(s) global, I. DOI: 10.1107/S2056989015012311/rk2431sup1.cif

Structure factors: contains datablock(s) I. DOI: 10.1107/S2056989015012311/rk2431Isup2.hkl

**Fig. 1.**
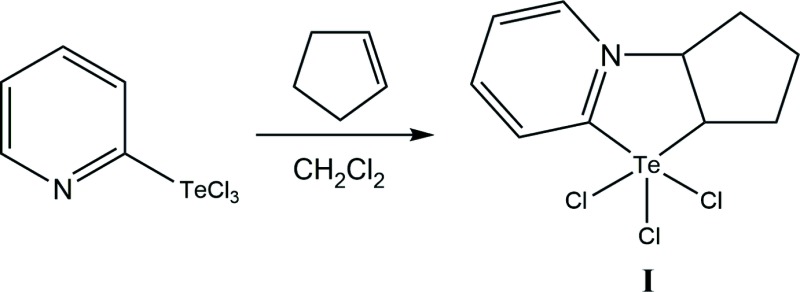
The reaction of 2–pyridinetellurium trichloride with cyclopentene.

. DOI: 10.1107/S2056989015012311/rk2431fig1.tif
The reaction of 2–pyridine­tellurium trichloride with cyclo­pentene.

**Fig. 2.**
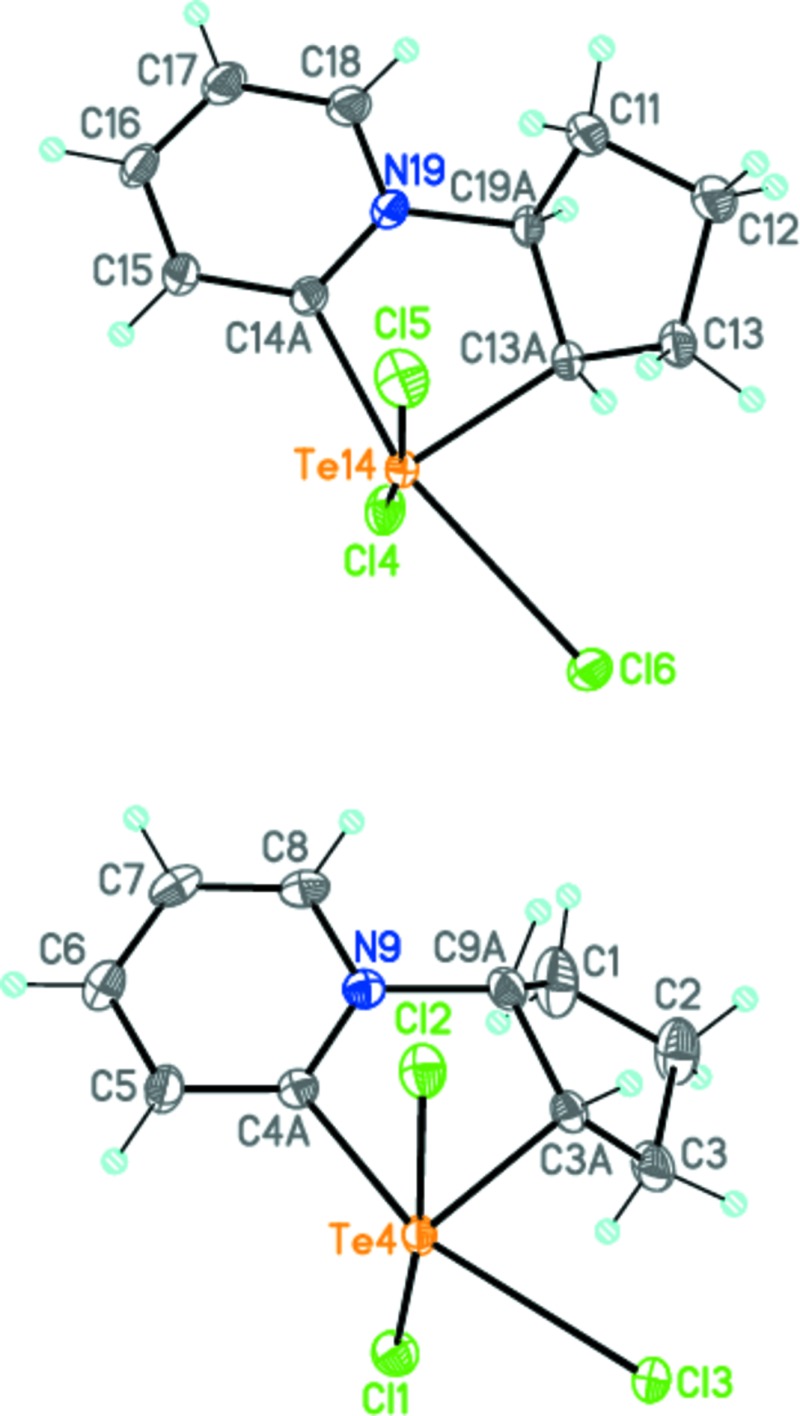
Molecular structure of (I) (the two crystallographically independent molecules are depicted). Displacement ellipsoids are shown at the 50% probability level. H atoms are presented as small spheres of arbitrary radius.

I . DOI: 10.1107/S2056989015012311/rk2431fig2.tif
Mol­ecular structure of (**I**) (the two crystallographically independent mol­ecules are depicted). Displacement ellipsoids are shown at the 50% probability level. H atoms are presented as small spheres of arbitrary radius.

**Fig. 3.**
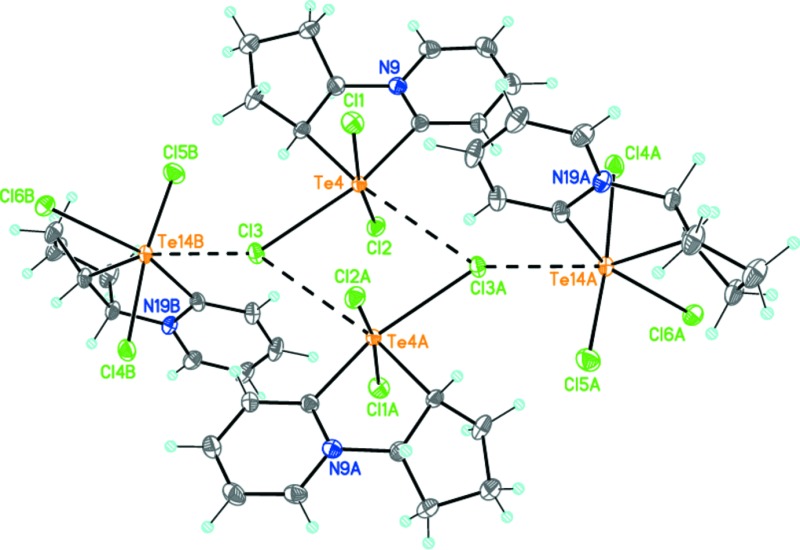
The centrosymmetrical 2+2–associates of (I). Dashed lines indicate the intermolecular non–valent attractive Te···Cl interactions.

I . DOI: 10.1107/S2056989015012311/rk2431fig3.tif
The centrosymmetrical 2 + 2–associates of (**I**). Dashed lines indicate the inter­molecular non–valent attractive Te⋯Cl inter­actions.

**Fig. 4.**
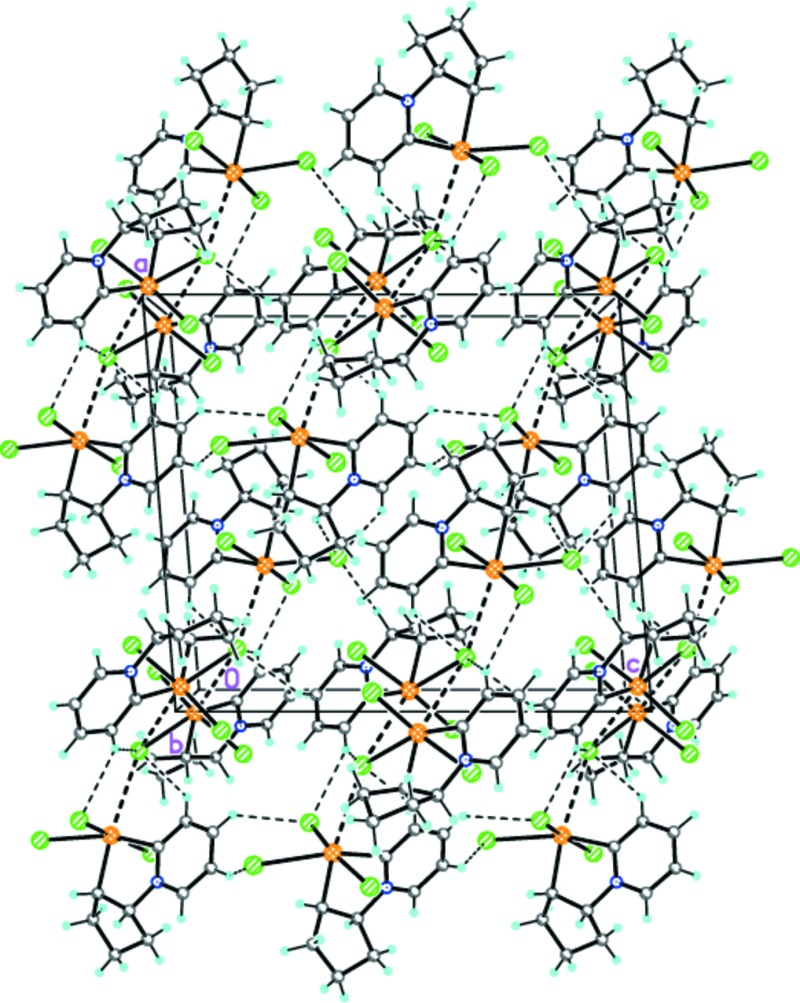
Crystal packing of (I). The thick dashed lines indicate the intermolecular non–valent attractive Te···Cl interactions, and the thin dashed lines indicate the intermolecular C—H···Cl hydrogen bonds.

I . DOI: 10.1107/S2056989015012311/rk2431fig4.tif
Crystal packing of (**I**). The thick dashed lines indicate the inter­molecular non–valent attractive Te⋯Cl inter­actions, and the thin dashed lines indicate the inter­molecular C—H⋯Cl hydrogen bonds.

CCDC reference: 1409052

Additional supporting information:  crystallographic information; 3D view; checkCIF report

## Figures and Tables

**Table 1:** Hydrogen-bond geometry (, )

*D*H*A*	*D*H	H*A*	*D* *A*	*D*H*A*
C5H5Cl3^i^	0.95	2.78	3.533(3)	137
C7H7Cl3^ii^	0.95	2.78	3.340(3)	119
C9*A*H9*A*Cl6	1.00	2.57	3.465(3)	149
C15H15Cl3^ii^	0.95	2.63	3.384(3)	136
C17H17Cl6^iii^	0.95	2.74	3.558(3)	145
C18H18Cl6^iv^	0.95	2.73	3.539(3)	144
C19*A*H19*A*Cl6^iv^	1.00	2.69	3.544(3)	144

Symmetry codes: (i) 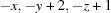; (ii) 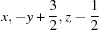; (iii) 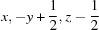; (iv) 
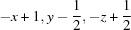
.

## supplementary crystallographic information

### S1. Structural commentary

It is known that the reaction of arenetellurium trihalides *Ar*Te*Hal*_3_ with alkenes and acetyl­enes usually gives the products of 1,2–addition at the multiple bonds, β–halo­alkyl­(vinyl)­tellurium dihalides or the products of transannular cyclization with the ring closure by the electron–donating center of the functional group containing in the molecule of the unsaturated substrate, lactones, ordinary cyclic ethers, pyrrolidine and piperidine derivatives (Petragnani & Stefani, 2007).

This work reports the structural characterization of a product of reaction of 2–pyridine­tellurium trichloride - the first representative of hetarenetellurium trihalides containing a nitro­gen base as the hetaryl substituent (Borisov *et al.*, 2013) with cyclo­pentene (Figure 1).

Compound (I), C_10_H_12_Cl_3_NTe, crystallizes with two crystallographically independent molecules in the asymmetric unit (Figure 2). These crystallographically independent molecules are geometrically very similar. The coordination around the tellurium atom is a distorted square–pyramidal. The equatorial plane is composed of the three chlorine atoms and the carbon atom of pyridinium ring. The tellurium atom is displaced from the mean square plane by 0.1926 (7) and 0.1981 (8) Å for the two crystallographically independent molecules, respectively, away from the apical carbon atom. The bond lengths from the tellurium atom to the two chlorine atoms arranged *trans* to each other [2.5009 (7)/2.5145 (7) and 2.5184 (7)/2.5220 (8) Å for the two crystallographically independent molecules, respectively] are close to those in related complexes (Singh *et al.*, 1990; Sundberg *et al.*, 1994; Zukerman–Schpector *et al.*, 2000; Kandasamy *et al.*, 2003; Raghavendra *et al.*, 2006; Dutton *et al.*, 2009; Lee *et al.*, 2010; Rakesh *et al.*, 2012). The third Te—Cl distance (2.8786 (7) and 2.8763 (7) Å for the two crystallographically independent molecules, respectively) is substanti­ally longer than the other two Te—Cl distances. This geometry is apparently determined by the zwitterionic nature of the complex and the hypervalent configuration of the tellurium atom. The analogous geometry was observed previously for tri­chloro­(ethane–1,2-diolato–*O*,*O*')tellurate(IV) (Sundberg *et al.*, 1994). The Te—C distances are in good agreement with typical values found in tellurium(IV) complexes, which range from 2.11 to 2.16 Å. The 1,3–tellurazole ring in (I) is almost planar (r.m.s. deviation is 0.042 and 0.045 Å for the two crystallographically independent molecules, respectively). The cyclo­pentane ring adopts the usual *envelope* conformation.

In the crystal, the molecules of (I) form centrosymmetrical 2+2–associates *via* additional non–valent attractive Te···Cl inter­actions (Te4···Cl3 [-*x*, 2-*y*, 1-*z*] 3.3993 (7) Å, Te14···Cl3 [*x*, 1.5-*y*, -0.5+*z*] 3.2030 (7) Å), in which the Cl3 chlorine atom is *µ*^3^–bridging, while the Cl6 chlorine atom is terminal (Figure 3). Due to these additional secondary inter­actions, the tellurium atom attains the strongly distorted 5+1–o­cta­hedral environment. Further, the 2+2–associates of (I) are bound by weak inter­molecular C—H···Cl hydrogen bonds into a 3–dimensional framework (Table 1, Figure 4). There are no inter­molecular Cl···Cl inter­actions.

The molecule of (I) possesses two asymmetric centers at the C3A and C9A carbon atoms and can have potentially four diastereomers. The crystal of (I) is racemic and consists of enanti­omeric pairs with the following relative configuration of the centers: *rac*-3A*R**,9A*S**.

### S2. Synthesis and crystallization

Complex (I) was prepared according to the procedure described by us earlier (Borisov *et al.*, 2013). The single crystals of (I) suitable for an X–ray diffraction analysis were obtained after recrystallization of the crude product from methyl­ene chloride.

### S3. Refinement

All hydrogen atoms were placed in calculated positions with C—H = 0.95 Å (for aryl–H), 0.99 Å (for methyl­ene–H) and 1.00 Å (for methine–H) and refined in the riding model with fixed isotropic displacement parameters [*U*_iso_(H) = 1.2*U*_eq_(C)].

### Crystal data

**Table:**

C_10_H_12_Cl_3_NTe	*F*(000) = 1456
*M**_r_* = 380.16	*D*_x_ = 1.928 Mg m^−^^3^
Monoclinic, *P*2_1_/*c*	Mo *K*α radiation, λ = 0.71073 Å
*a* = 14.3279 (6) Å	Cell parameters from 9992 reflections
*b* = 11.2539 (5) Å	θ = 2.2–32.5°
*c* = 16.2967 (7) Å	µ = 2.85 mm^−^^1^
β = 94.546 (1)°	*T* = 120 K
*V* = 2619.5 (2) Å^3^	Prism, yellow
*Z* = 8	0.20 × 0.15 × 0.15 mm

### Data collection

**Table:**

Bruker APEXII CCD diffractometer	6535 reflections with *I* > 2σ(*I*)
Radiation source: fine–focus sealed tube	*R*_int_ = 0.037
φ and ω scans	θ_max_ = 30.0°, θ_min_ = 1.4°
Absorption correction: multi-scan (*SADABS*; Bruker, 2003)	*h* = −20→20
*T*_min_ = 0.595, *T*_max_ = 0.666	*k* = −15→15
32448 measured reflections	*l* = −22→22
7642 independent reflections	

### Refinement

**Table:**

Refinement on *F*^2^	Primary atom site location: structure-invariant direct methods
Least-squares matrix: full	Secondary atom site location: difference Fourier map
*R*[*F*^2^ > 2σ(*F*^2^)] = 0.030	Hydrogen site location: inferred from neighbouring sites
*wR*(*F*^2^) = 0.059	H-atom parameters constrained
*S* = 1.08	*w* = 1/[σ^2^(*F*_o_^2^) + (0.0215*P*)^2^ + 1.2054*P*] where *P* = (*F*_o_^2^ + 2*F*_c_^2^)/3
7642 reflections	(Δ/σ)_max_ = 0.001
271 parameters	Δρ_max_ = 0.76 e Å^−^^3^
0 restraints	Δρ_min_ = −0.56 e Å^−^^3^

### Special details

**Table:**

Geometry. All s.u.'s (except the s.u. in the dihedral angle between two l.s. planes) are estimated using the full covariance matrix. The cell s.u.'s are taken into account individually in the estimation of s.u.'s in distances, angles and torsion angles; correlations between s.u.'s in cell parameters are only used when they are defined by crystal symmetry. An approximate (isotropic) treatment of cell s.u.'s is used for estimating s.u.'s involving l.s. planes.

### Fractional atomic coordinates and isotropic or equivalent isotropic displacement parameters (Å^2^)

**Table:**

	*x*	*y*	*z*	*U*_iso_*/*U*_eq_	
Cl1	−0.05985 (5)	0.72835 (7)	0.58167 (5)	0.02922 (15)	
Cl2	0.14184 (5)	0.93448 (6)	0.38971 (4)	0.02747 (15)	
Cl3	0.12938 (5)	1.00286 (6)	0.61789 (4)	0.02427 (14)	
C1	0.1831 (3)	0.5277 (3)	0.5165 (2)	0.0453 (9)	
H1A	0.1220	0.4886	0.5215	0.054*	
H1B	0.2254	0.4714	0.4911	0.054*	
C2	0.2250 (3)	0.5689 (3)	0.5989 (2)	0.0422 (9)	
H2A	0.2929	0.5843	0.5976	0.051*	
H2B	0.2155	0.5091	0.6420	0.051*	
C3	0.1724 (2)	0.6828 (3)	0.61445 (19)	0.0329 (7)	
H3A	0.2083	0.7337	0.6552	0.040*	
H3B	0.1101	0.6658	0.6341	0.040*	
C3A	0.16345 (18)	0.7410 (2)	0.52939 (17)	0.0214 (5)	
H3C	0.2194	0.7934	0.5259	0.026*	
Te4	0.04220 (2)	0.84673 (2)	0.49461 (2)	0.01829 (5)	
C4A	0.01838 (19)	0.7106 (2)	0.40406 (17)	0.0213 (5)	
C5	−0.0573 (2)	0.7027 (3)	0.34626 (18)	0.0275 (6)	
H5	−0.1078	0.7574	0.3471	0.033*	
C6	−0.0589 (2)	0.6143 (3)	0.28727 (18)	0.0311 (7)	
H6	−0.1106	0.6076	0.2473	0.037*	
C7	0.0154 (2)	0.5356 (3)	0.28687 (18)	0.0313 (7)	
H7	0.0149	0.4743	0.2468	0.038*	
C8	0.0895 (2)	0.5471 (3)	0.34498 (18)	0.0272 (6)	
H8	0.1413	0.4945	0.3444	0.033*	
N9	0.08939 (16)	0.6327 (2)	0.40306 (14)	0.0219 (5)	
C9A	0.1713 (2)	0.6402 (3)	0.46615 (19)	0.0265 (6)	
H9A	0.2293	0.6531	0.4370	0.032*	
Cl4	0.28653 (5)	0.27629 (7)	0.24383 (4)	0.02950 (16)	
Cl5	0.40398 (6)	0.64964 (7)	0.11901 (5)	0.03456 (17)	
Cl6	0.36234 (5)	0.56379 (6)	0.35946 (4)	0.02687 (15)	
C11	0.5970 (2)	0.4070 (3)	0.1259 (2)	0.0338 (7)	
H11A	0.6415	0.3549	0.0995	0.041*	
H11B	0.5744	0.4696	0.0865	0.041*	
C12	0.6412 (2)	0.4601 (3)	0.2044 (2)	0.0369 (8)	
H12A	0.6820	0.5280	0.1927	0.044*	
H12B	0.6787	0.4001	0.2369	0.044*	
C13	0.55749 (19)	0.5014 (3)	0.2505 (2)	0.0306 (7)	
H13A	0.5737	0.5025	0.3107	0.037*	
H13B	0.5368	0.5818	0.2325	0.037*	
C13A	0.48125 (18)	0.4089 (3)	0.22756 (17)	0.0220 (6)	
H13C	0.4777	0.3543	0.2756	0.026*	
Te14	0.34174 (2)	0.47397 (2)	0.19378 (2)	0.01873 (5)	
C14A	0.36030 (19)	0.3737 (3)	0.08412 (16)	0.0217 (6)	
C15	0.2958 (2)	0.3570 (3)	0.01782 (17)	0.0262 (6)	
H15	0.2388	0.4004	0.0138	0.031*	
C16	0.3149 (2)	0.2764 (3)	−0.04306 (17)	0.0307 (7)	
H16	0.2713	0.2653	−0.0894	0.037*	
C17	0.3974 (2)	0.2124 (3)	−0.03626 (19)	0.0319 (7)	
H17	0.4099	0.1551	−0.0768	0.038*	
C18	0.4612 (2)	0.2323 (3)	0.02985 (18)	0.0276 (6)	
H18	0.5186	0.1898	0.0347	0.033*	
N19	0.44226 (16)	0.3125 (2)	0.08791 (14)	0.0222 (5)	
C19A	0.51593 (18)	0.3360 (3)	0.15587 (17)	0.0234 (6)	
H19A	0.5405	0.2582	0.1779	0.028*	

### Atomic displacement parameters (Å^2^)

**Table:**

	*U*^11^	*U*^22^	*U*^33^	*U*^12^	*U*^13^	*U*^23^
Cl1	0.0258 (3)	0.0311 (4)	0.0313 (4)	−0.0041 (3)	0.0053 (3)	−0.0004 (3)
Cl2	0.0271 (3)	0.0286 (4)	0.0263 (4)	−0.0033 (3)	−0.0006 (3)	0.0069 (3)
Cl3	0.0208 (3)	0.0290 (4)	0.0222 (3)	−0.0004 (3)	−0.0029 (2)	−0.0050 (3)
C1	0.051 (2)	0.0229 (16)	0.057 (2)	0.0052 (15)	−0.0242 (18)	0.0049 (16)
C2	0.048 (2)	0.0284 (17)	0.047 (2)	0.0007 (15)	−0.0177 (17)	0.0069 (15)
C3	0.0283 (16)	0.0419 (19)	0.0277 (16)	0.0102 (14)	−0.0034 (12)	0.0090 (14)
C3A	0.0181 (12)	0.0208 (13)	0.0247 (14)	0.0035 (10)	−0.0017 (10)	0.0023 (11)
Te4	0.01718 (8)	0.01745 (8)	0.01955 (9)	0.00076 (6)	−0.00290 (6)	−0.00165 (7)
C4A	0.0222 (13)	0.0208 (13)	0.0209 (13)	0.0001 (11)	0.0021 (10)	−0.0005 (11)
C5	0.0299 (15)	0.0264 (15)	0.0248 (15)	0.0027 (12)	−0.0067 (12)	−0.0029 (12)
C6	0.0371 (17)	0.0318 (16)	0.0233 (15)	−0.0023 (13)	−0.0037 (13)	−0.0031 (13)
C7	0.0435 (18)	0.0300 (16)	0.0212 (15)	−0.0057 (14)	0.0084 (13)	−0.0071 (13)
C8	0.0335 (16)	0.0227 (14)	0.0263 (15)	0.0031 (12)	0.0084 (12)	−0.0029 (12)
N9	0.0224 (11)	0.0212 (12)	0.0223 (12)	−0.0008 (9)	0.0032 (9)	−0.0002 (9)
C9A	0.0214 (14)	0.0258 (15)	0.0317 (16)	0.0061 (11)	−0.0027 (12)	−0.0038 (12)
Cl4	0.0306 (4)	0.0338 (4)	0.0233 (3)	−0.0059 (3)	−0.0024 (3)	0.0080 (3)
Cl5	0.0336 (4)	0.0306 (4)	0.0393 (4)	−0.0030 (3)	0.0012 (3)	0.0096 (3)
Cl6	0.0283 (4)	0.0286 (4)	0.0239 (3)	−0.0009 (3)	0.0033 (3)	−0.0047 (3)
C11	0.0202 (14)	0.050 (2)	0.0319 (17)	−0.0041 (14)	0.0028 (12)	−0.0112 (15)
C12	0.0240 (15)	0.048 (2)	0.0391 (19)	−0.0058 (14)	0.0030 (13)	−0.0151 (16)
C13	0.0201 (14)	0.0386 (18)	0.0321 (17)	0.0015 (12)	−0.0044 (12)	−0.0116 (14)
C13A	0.0165 (12)	0.0299 (15)	0.0190 (13)	0.0028 (11)	−0.0023 (10)	−0.0020 (11)
Te14	0.01635 (8)	0.02350 (9)	0.01601 (8)	0.00111 (7)	−0.00087 (6)	0.00012 (7)
C14A	0.0215 (13)	0.0270 (15)	0.0165 (13)	−0.0005 (11)	0.0016 (10)	0.0042 (11)
C15	0.0227 (14)	0.0365 (17)	0.0189 (14)	−0.0001 (12)	−0.0007 (11)	0.0016 (12)
C16	0.0292 (15)	0.047 (2)	0.0154 (13)	−0.0090 (14)	−0.0004 (11)	−0.0029 (13)
C17	0.0330 (16)	0.0387 (18)	0.0241 (15)	−0.0061 (14)	0.0030 (12)	−0.0100 (13)
C18	0.0284 (15)	0.0295 (16)	0.0254 (15)	−0.0015 (12)	0.0047 (12)	−0.0066 (12)
N19	0.0227 (12)	0.0262 (12)	0.0175 (11)	−0.0017 (9)	0.0004 (9)	−0.0030 (9)
C19A	0.0186 (13)	0.0282 (15)	0.0222 (14)	0.0027 (11)	−0.0045 (10)	−0.0062 (12)

### Geometric parameters (Å, º)

**Table:**

Cl1—Te4	2.5009 (7)	Cl4—Te14	2.5184 (7)
Cl2—Te4	2.5145 (7)	Cl5—Te14	2.5220 (8)
Cl3—Te4	2.8786 (7)	Cl6—Te14	2.8763 (7)
C1—C2	1.501 (5)	C11—C12	1.506 (4)
C1—C9A	1.510 (4)	C11—C19A	1.521 (4)
C1—H1A	0.9900	C11—H11A	0.9900
C1—H1B	0.9900	C11—H11B	0.9900
C2—C3	1.518 (5)	C12—C13	1.536 (4)
C2—H2A	0.9900	C12—H12A	0.9900
C2—H2B	0.9900	C12—H12B	0.9900
C3—C3A	1.529 (4)	C13—C13A	1.533 (4)
C3—H3A	0.9900	C13—H13A	0.9900
C3—H3B	0.9900	C13—H13B	0.9900
C3A—C9A	1.543 (4)	C13A—C19A	1.542 (4)
C3A—Te4	2.144 (3)	C13A—Te14	2.159 (3)
C3A—H3C	1.0000	C13A—H13C	1.0000
Te4—C4A	2.136 (3)	Te14—C14A	2.148 (3)
C4A—N9	1.344 (3)	C14A—N19	1.359 (3)
C4A—C5	1.382 (4)	C14A—C15	1.377 (4)
C5—C6	1.382 (4)	C15—C16	1.388 (4)
C5—H5	0.9500	C15—H15	0.9500
C6—C7	1.385 (4)	C16—C17	1.381 (4)
C6—H6	0.9500	C16—H16	0.9500
C7—C8	1.372 (4)	C17—C18	1.375 (4)
C7—H7	0.9500	C17—H17	0.9500
C8—N9	1.351 (4)	C18—N19	1.350 (4)
C8—H8	0.9500	C18—H18	0.9500
N9—C9A	1.500 (4)	N19—C19A	1.492 (3)
C9A—H9A	1.0000	C19A—H19A	1.0000
			
C2—C1—C9A	104.3 (3)	C12—C11—C19A	102.5 (2)
C2—C1—H1A	110.9	C12—C11—H11A	111.3
C9A—C1—H1A	110.9	C19A—C11—H11A	111.3
C2—C1—H1B	110.9	C12—C11—H11B	111.3
C9A—C1—H1B	110.9	C19A—C11—H11B	111.3
H1A—C1—H1B	108.9	H11A—C11—H11B	109.2
C1—C2—C3	104.0 (3)	C11—C12—C13	104.1 (2)
C1—C2—H2A	111.0	C11—C12—H12A	110.9
C3—C2—H2A	111.0	C13—C12—H12A	110.9
C1—C2—H2B	111.0	C11—C12—H12B	110.9
C3—C2—H2B	111.0	C13—C12—H12B	110.9
H2A—C2—H2B	109.0	H12A—C12—H12B	109.0
C2—C3—C3A	102.6 (3)	C13A—C13—C12	104.1 (2)
C2—C3—H3A	111.3	C13A—C13—H13A	110.9
C3A—C3—H3A	111.3	C12—C13—H13A	110.9
C2—C3—H3B	111.3	C13A—C13—H13B	110.9
C3A—C3—H3B	111.3	C12—C13—H13B	110.9
H3A—C3—H3B	109.2	H13A—C13—H13B	109.0
C3—C3A—C9A	106.6 (2)	C13—C13A—C19A	106.3 (2)
C3—C3A—Te4	119.02 (19)	C13—C13A—Te14	117.36 (19)
C9A—C3A—Te4	109.39 (17)	C19A—C13A—Te14	109.55 (17)
C3—C3A—H3C	107.1	C13—C13A—H13C	107.8
C9A—C3A—H3C	107.1	C19A—C13A—H13C	107.8
Te4—C3A—H3C	107.1	Te14—C13A—H13C	107.8
C4A—Te4—C3A	82.32 (10)	C14A—Te14—C13A	82.00 (10)
C4A—Te4—Cl1	86.64 (8)	C14A—Te14—Cl4	82.42 (7)
C3A—Te4—Cl1	93.00 (8)	C13A—Te14—Cl4	85.77 (8)
C4A—Te4—Cl2	83.11 (8)	C14A—Te14—Cl5	86.45 (8)
C3A—Te4—Cl2	84.59 (8)	C13A—Te14—Cl5	91.91 (8)
Cl1—Te4—Cl2	169.70 (3)	Cl4—Te14—Cl5	168.84 (3)
N9—C4A—C5	120.2 (3)	N19—C14A—C15	119.4 (3)
N9—C4A—Te4	113.36 (19)	N19—C14A—Te14	113.09 (18)
C5—C4A—Te4	126.2 (2)	C15—C14A—Te14	127.2 (2)
C4A—C5—C6	119.3 (3)	C14A—C15—C16	119.4 (3)
C4A—C5—H5	120.4	C14A—C15—H15	120.3
C6—C5—H5	120.4	C16—C15—H15	120.3
C5—C6—C7	119.6 (3)	C17—C16—C15	120.0 (3)
C5—C6—H6	120.2	C17—C16—H16	120.0
C7—C6—H6	120.2	C15—C16—H16	120.0
C8—C7—C6	119.3 (3)	C18—C17—C16	119.3 (3)
C8—C7—H7	120.3	C18—C17—H17	120.4
C6—C7—H7	120.3	C16—C17—H17	120.4
N9—C8—C7	120.4 (3)	N19—C18—C17	120.0 (3)
N9—C8—H8	119.8	N19—C18—H18	120.0
C7—C8—H8	119.8	C17—C18—H18	120.0
C4A—N9—C8	121.1 (2)	C18—N19—C14A	121.8 (2)
C4A—N9—C9A	120.5 (2)	C18—N19—C19A	118.0 (2)
C8—N9—C9A	118.4 (2)	C14A—N19—C19A	120.1 (2)
N9—C9A—C1	111.9 (2)	N19—C19A—C11	111.6 (2)
N9—C9A—C3A	113.9 (2)	N19—C19A—C13A	113.9 (2)
C1—C9A—C3A	105.3 (3)	C11—C19A—C13A	105.3 (2)
N9—C9A—H9A	108.5	N19—C19A—H19A	108.6
C1—C9A—H9A	108.5	C11—C19A—H19A	108.6
C3A—C9A—H9A	108.5	C13A—C19A—H19A	108.6
			
C9A—C1—C2—C3	40.7 (4)	C19A—C11—C12—C13	−42.7 (3)
C1—C2—C3—C3A	−39.5 (4)	C11—C12—C13—C13A	34.0 (3)
C2—C3—C3A—C9A	23.5 (3)	C12—C13—C13A—C19A	−12.0 (3)
C2—C3—C3A—Te4	147.7 (2)	C12—C13—C13A—Te14	−134.9 (2)
N9—C4A—C5—C6	−0.4 (4)	N19—C14A—C15—C16	1.2 (4)
Te4—C4A—C5—C6	174.7 (2)	Te14—C14A—C15—C16	−171.3 (2)
C4A—C5—C6—C7	−0.2 (5)	C14A—C15—C16—C17	1.0 (5)
C5—C6—C7—C8	−0.3 (5)	C15—C16—C17—C18	−2.1 (5)
C6—C7—C8—N9	1.3 (5)	C16—C17—C18—N19	1.1 (5)
C5—C4A—N9—C8	1.5 (4)	C17—C18—N19—C14A	1.2 (4)
Te4—C4A—N9—C8	−174.2 (2)	C17—C18—N19—C19A	−176.6 (3)
C5—C4A—N9—C9A	−179.4 (3)	C15—C14A—N19—C18	−2.4 (4)
Te4—C4A—N9—C9A	4.9 (3)	Te14—C14A—N19—C18	171.2 (2)
C7—C8—N9—C4A	−2.0 (4)	C15—C14A—N19—C19A	175.4 (3)
C7—C8—N9—C9A	178.9 (3)	Te14—C14A—N19—C19A	−11.1 (3)
C4A—N9—C9A—C1	120.3 (3)	C18—N19—C19A—C11	72.4 (3)
C8—N9—C9A—C1	−60.6 (4)	C14A—N19—C19A—C11	−105.4 (3)
C4A—N9—C9A—C3A	1.0 (4)	C18—N19—C19A—C13A	−168.5 (3)
C8—N9—C9A—C3A	−179.9 (2)	C14A—N19—C19A—C13A	13.7 (4)
C2—C1—C9A—N9	−149.5 (3)	C12—C11—C19A—N19	158.9 (2)
C2—C1—C9A—C3A	−25.2 (4)	C12—C11—C19A—C13A	34.8 (3)
C3—C3A—C9A—N9	123.7 (3)	C13—C13A—C19A—N19	−136.5 (2)
Te4—C3A—C9A—N9	−6.2 (3)	Te14—C13A—C19A—N19	−8.8 (3)
C3—C3A—C9A—C1	0.8 (3)	C13—C13A—C19A—C11	−13.9 (3)
Te4—C3A—C9A—C1	−129.1 (2)	Te14—C13A—C19A—C11	113.8 (2)

### Hydrogen-bond geometry (Å, º)

**Table:**

*D*—H···*A*	*D*—H	H···*A*	*D*···*A*	*D*—H···*A*
C5—H5···Cl3^i^	0.95	2.78	3.533 (3)	137
C7—H7···Cl3^ii^	0.95	2.78	3.340 (3)	119
C9*A*—H9*A*···Cl6	1.00	2.57	3.465 (3)	149
C15—H15···Cl3^ii^	0.95	2.63	3.384 (3)	136
C17—H17···Cl6^iii^	0.95	2.74	3.558 (3)	145
C18—H18···Cl6^iv^	0.95	2.73	3.539 (3)	144
C19*A*—H19*A*···Cl6^iv^	1.00	2.69	3.544 (3)	144

Symmetry codes: (i) −*x*, −*y*+2, −*z*+1; (ii) *x*, −*y*+3/2, *z*−1/2; (iii) *x*, −*y*+1/2, *z*−1/2; (iv) −*x*+1, *y*−1/2, −*z*+1/2.
