# Supplementary figures and images for: Crystal structure of rac-(3aR*,9aS*)-4,4,4-tri­chloro-1,2,3,3a,4,9a-hexa­hydro-4λ5,9λ4-cyclo­penta­[4,5][1,3]tellurazolo[3,2-a]pyridine
Source: Acta Crystallogr E Crystallogr Commun. 2015 Jul 22;71(Pt 8):o598–9. doi: 10.1107/S2056989015012311 (PMC4571417; doi:10.1107/S2056989015012311)

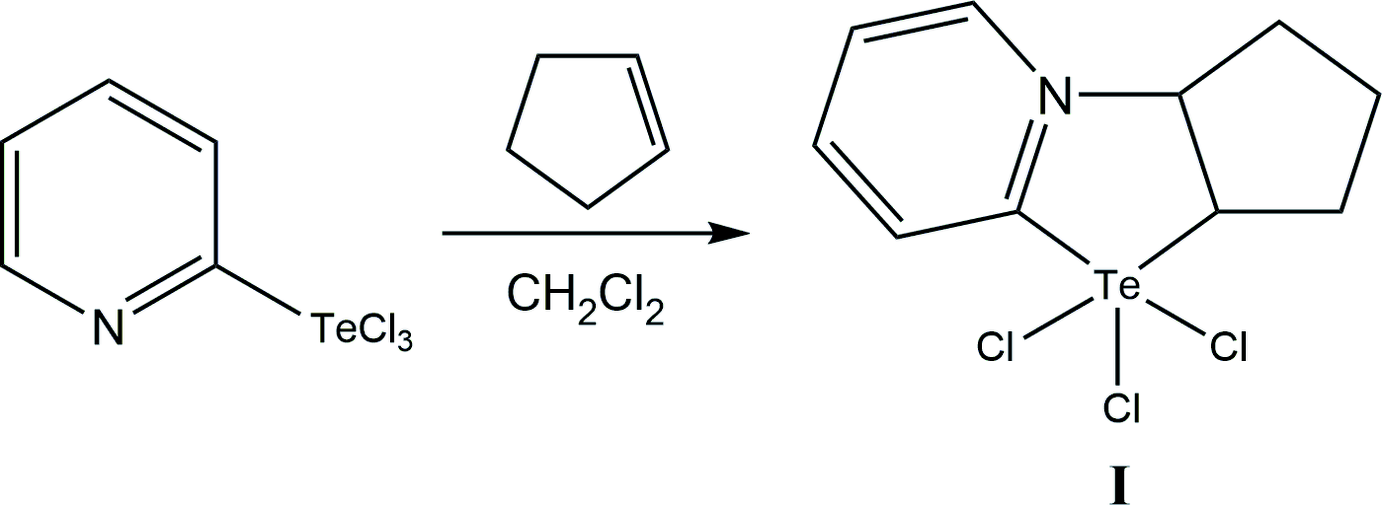

Supplement: Supplementary file 3 [file e-71-0o598-fig1.tif]

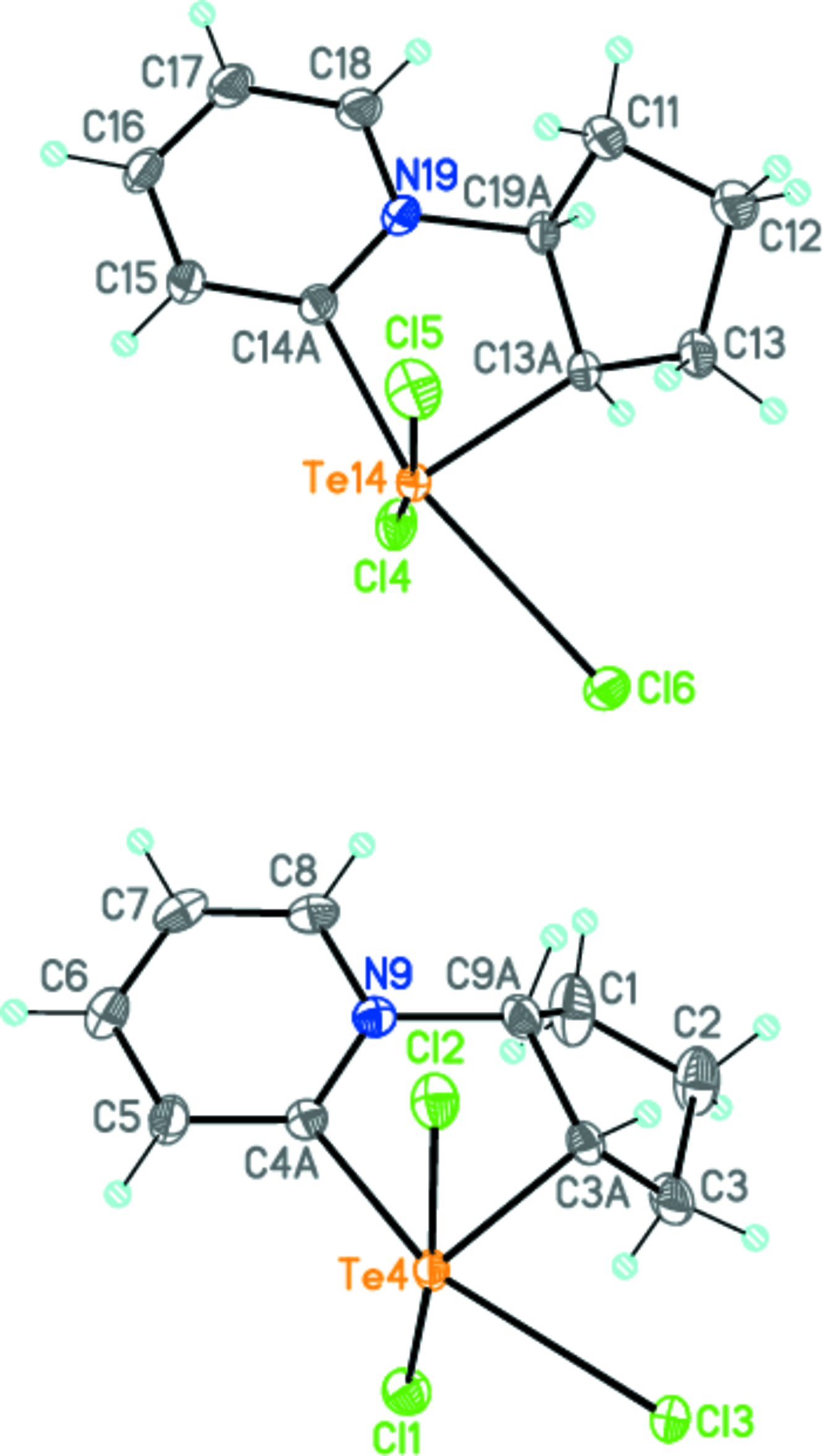

Supplement: Supplementary file 4 [file e-71-0o598-fig2.tif]

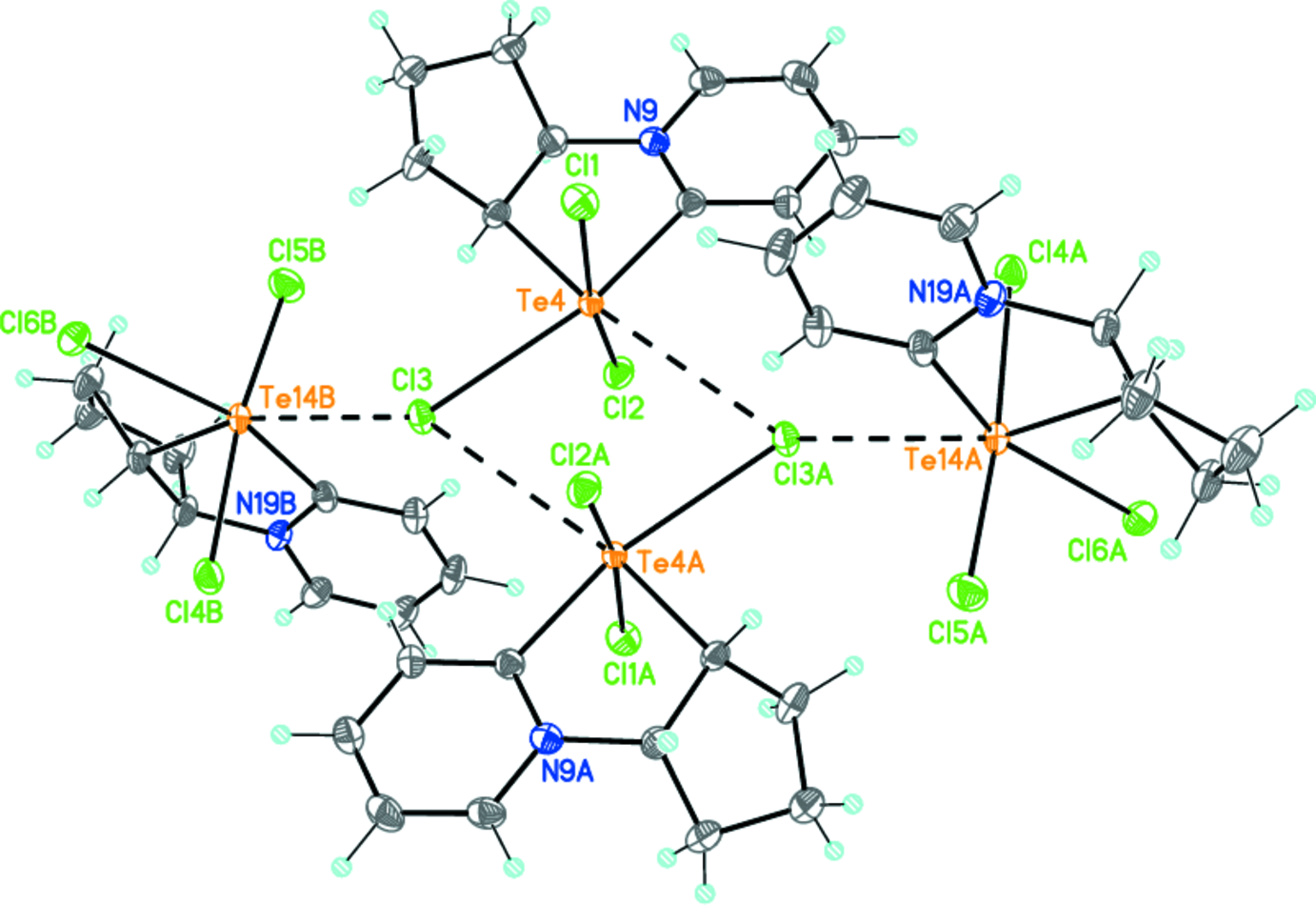

Supplement: Supplementary file 5 [file e-71-0o598-fig3.tif]

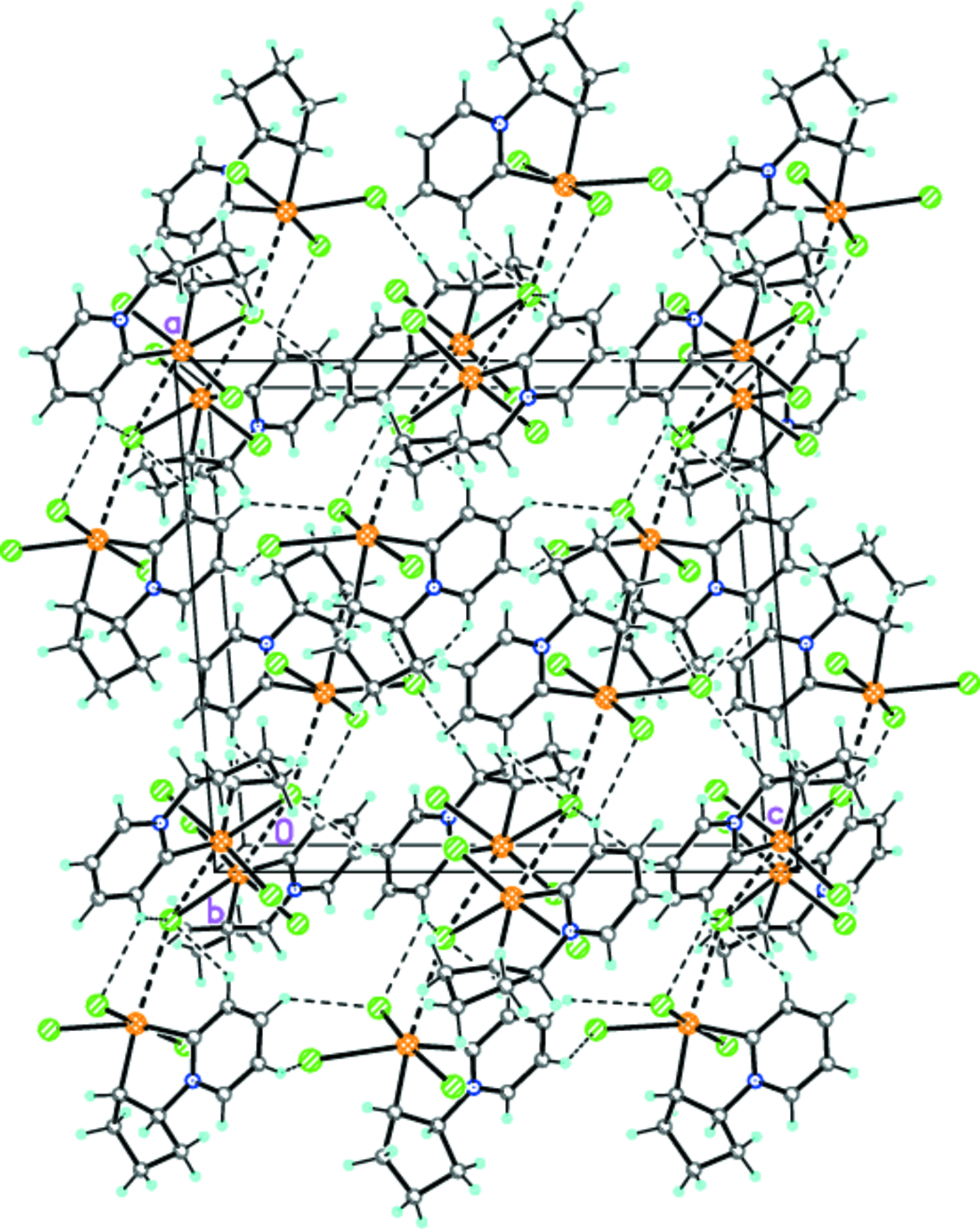

Supplement: Supplementary file 6 [file e-71-0o598-fig4.tif]
